# The FH mutation database: an online database of fumarate hydratase mutations involved in the MCUL (HLRCC) tumor syndrome and congenital fumarase deficiency

**DOI:** 10.1186/1471-2350-9-20

**Published:** 2008-03-25

**Authors:** Jean-Pierre Bayley, Virpi Launonen, Ian PM Tomlinson

**Affiliations:** 1 Department of Human Genetics, Leiden University Medical Center, P.O. Box 9503, 2300 RA, Leiden, The Netherlands; 2 Department of Medical Genetics, University of Helsinki, Biomedicum Helsinki, Haartmaninkatu 8, Finland; 3 Molecular and Population Genetics Laboratory, Cancer Research UK, 44, Lincoln's Inn Fields, London WC2A 3PX, UK

## Abstract

**Background:**

Fumarate hydratase (HGNC approved gene symbol – *FH*), also known as fumarase, is an enzyme of the tricarboxylic acid (TCA) cycle, involved in fundamental cellular energy production. First described by Zinn *et al *in 1986, deficiency of FH results in early onset, severe encephalopathy. In 2002, the Multiple Leiomyoma Consortium identified heterozygous germline mutations of *FH *in patients with multiple cutaneous and uterine leiomyomas, (MCUL: OMIM 150800). In some families renal cell cancer also forms a component of the complex and as such has been described as hereditary leiomyomatosis and renal cell cancer (HLRCC: OMIM 605839). The identification of FH as a tumor suppressor was an unexpected finding and following the identification of subunits of succinate dehydrogenase in 2000 and 2001, was only the second description of the involvement of an enzyme of intermediary metabolism in tumorigenesis.

**Description:**

The *FH *mutation database is a part of the TCA cycle gene mutation database (formerly the succinate dehydrogenase gene mutation database) and is based on the Leiden Open (source) Variation Database (LOVD) system. The variants included in the database were derived from the published literature and annotated to conform to current mutation nomenclature. The *FH *database applies HGVS nomenclature guidelines, and will assist researchers in applying these guidelines when directly submitting new sequence variants online. Since the first molecular characterization of an *FH *mutation by Bourgeron *et al *in 1994, a series of reports of both FH deficiency patients and patients with MCUL/HLRRC have described 107 variants, of which 93 are thought to be pathogenic. The most common type of mutation is missense (57%), followed by frameshifts & nonsense (27%), and diverse deletions, insertions and duplications. Here we introduce an online database detailing all reported *FH *sequence variants.

**Conclusion:**

The *FH *mutation database strives to systematically unify all current genetic knowledge of *FH *variants. We believe that this knowledge will assist clinical geneticists and treating physicians when advising patients and their families, will provide a rapid and convenient resource for research scientists, and may eventually assist in gaining novel insights into FH and its related clinical syndromes.

## Background

Recently two proteins involved in the tricarboxcylic acid (TCA) cycle have been shown to be tumor suppressors. Fumarate hydratase (FH) (also known as fumarase) and succinate dehydrogenase (SDH), which also plays a role in oxidative phosphorylation, are enzymes involved in fundamental processes of energy production. Deficiencies of FH and SDH(A) generally result in early-onset, severe encephalopathy. The first description of fumarate hydratase deficiency was in 1986 by Zinn *et al *[1], followed in 1994 by the first molecular characterization of an *FH *mutation by Bourgeron *et al *[2]. In 2002, the Multiple Leiomyoma Consortium identified *FH *as the tumor suppressor gene responsible for MCUL/HLRCC [3].

The identification of these genes as tumor suppressors was an entirely unexpected finding and demonstrated for the first time the involvement of proteins of intermediary metabolism in tumorigenesis. Mutations have been identified in both the gene encoding fumarate hydratase and three of the four genes encoding succinate dehydrogenase, subunits B, C and D (*SDHB, -C *and *-D*) [4-6], while no cancer-related mutations have yet been reported in *SDHA*. Germline mutations in *FH *predispose individuals to multiple cutaneous leiomyomas, uterine leiomyomas and in some families renal cell cancer (HLRCC) [7], whereas mutations in SDH cause hereditary paragangliomas and pheochromocytomas (HPGL) [8]. Both of these cancer syndromes are inherited in an autosomal dominant manner. Despite the closely related function of FH and SDH proteins, the tumor spectra in HPGL and HLRCC show little overlap, indicating that although biochemically related, the mitogenic stimulus leading to tumor formation must be cell specific.

The principal phenotype of the FH-associated tumor syndrome is skin leiomyoma. These are typically sensitive to cold or abrasion, appear to be more common in women than men, developing between the second and fourth decades as intradermal papules or nodules of up to 20 mm in diameter, with a disseminated or segmental distribution. Germline *FH *mutations have been identified in the vast majority of patients with multiple skin leiomyomas, and the relatives of probands have often been subsequently diagnosed with skin leiomyomas, suggesting that many more cases are currently going unrecognized. Female *FH *mutation carriers are also at high risk of early-onset uterine fibroids that frequently require hysterectomy. Certain *FH *mutations have also been associated with uterine fibroids without skin leiomyomas [9]. Although uterine fibroids are the most common tumors in women during their reproductive years, *FH *mutations do not appear to play a major role in non-syndromic cases [10]. Although not always present in the FH syndrome, aggressive renal cell carcinomas of two unusual types, type II papillary and collecting duct morphology, also occur in certain families. Both frequently present with metastatic disease before the age of fifty and are associated with high mortality. A single case has also been reported with both papillary and conventional clear cell renal carcinoma, both tumors displaying loss of the wild type *FH *allele and immunostaining [11]. Recently Merino *et al *described a distinctive histological feature of these tumors, a characteristically large nucleus with a very prominent inclusion-like orangiophilic or eosinophilic nucleolus, surrounded by a clear halo [12].

Leiomyosarcomas also occur in association with *FH *mutations but appear to be rare in FH mutation carriers although a few cases, predominantly of Finnish origin, have been described [13,14]. Other reported pathology associated with *FH *mutations include cerebral cavernomas [15] and Leydig cell tumors [16] and ovarian mucinous cystadenoma [17].

A number of biochemical studies have shown that FH-deficient cells and tumors accumulate both fumarate and succinate, while SDH-deficient tumors mainly accumulate succinate [18-20]. Accumulation of these metabolic intermediates may affect other biochemical processes in the cell, and considerable evidence indicates that the inhibition of HIF prolyl hydroxylase leads to the over-expression of the transcription factor, hypoxia-inducible factor 1α (HIF1α) [21]. HIF1α targets, including vascular endothelial growth factor (VEGF), erythropoietin (EPO), and glucose transporter1 (GLUT1), show activation on accumulation of TCA metabolites, and contribute to an increase in microvessel density [18,20,22-26]. This phenomenon has been described as "pseudo-hypoxia" and shows striking similarities with von Hippel-Lindau (VHL) syndrome [27]. In normoxia, HIF1α is hydroxylated at critical proline residues by a HIF prolyl hydroxylase, and targeted for degradation by an E3-ubiquitin ligase complex that includes the VHL protein. In VHL disease, mutations in *VHL *inhibit this process and lead to the stabilization of HIF1α. Phenotypically, both HPGL and VHL patients may develop pheochromocytomas, and the clear cell renal carcinomas that are a major feature of VHL disease [28] have recently been reported in a HPGL family [29].

## Construction and content

The *FH *mutation database [30] is based on the recently described Leiden Open (source) Variation Database (LOVD) system [31] which, under the auspices of the Human Genome Variation Society, is rapidly becoming the standard for gene-specific databases. The LOVD system was designed to be flexible so that it could be easily adapted to the needs of a wide range of locus specific databases. Additional flexibility comes from the platform-independent design and the use of PHP and MySQL open source software. The design is gene-centered and modular, and focuses on the collection and display of DNA sequence variations. The LOVD platform is also easily extendable to include summary clinical data. In addition, all database contents may be downloaded in flat text format and imported into a spreadsheet program for further analysis. The LOVD database software is now in a new version, LOVD2, which has many additional features.

Researchers interested in contributing sequence variants to the database will be asked to register, (submitters can access and edit their personal data at any time), after which they can contribute the variant online. During submission researchers are asked to provide those data that are deemed absolutely essential for mutation databases by the Human Genome Organization Mutation Database Initiative. These include a patient ID, an exact molecular description of the variant (DNA-level), and details about the source of the material and detection method used. Newly submitted variants are given a unique identifier, as recommended by Claustres *et al *[32]. After the curator's approval the new variant is added to the database and all connected web pages are updated instantly. Currently (Jan, 2008) all database content is derived from published, peer-reviewed literature. We anticipate that directly submitted content will become an increasing proportion in the future. As the submitted data are in the form of a submitter deduced mutation description, the curators can only check the feasibility and consistency of the description and not any original data.

DNA variation in populations is generally benign and as such should be termed a 'variant' or 'SNP'. The term 'mutation' is reserved for cases were there is a reasonable suspicion that the variant is deleterious. DNA variants submitted to the *FH *database should be described in accordance with the recommendations of the Human Genome Variation Society (HGVS), update August 2004, and it is strongly recommended that authors consult the most up-to-date nomenclature guidelines, which can be found online at the HGVS website. Unfortunately the mutation nomenclature currently used in the field of *FH *research is rarely in agreement with HGVS guidelines. Reports contain a variety of annotations, and many are based on the annotation of the cytosolic protein, the function of which is currently poorly defined [33]. One of the aims of the HGVS is to establish a consistent nomenclature across all human genes. The description of FH protein variants based on the cytosolic protein is often accompanied by the use of a truncated transcript to describe cDNA variants. Other authors use the cytosolic protein numbering system, while applying the full-length coding transcript to describe cDNA variants. This results in the loss of the simple approximation of 3 to 1 between the numbering. The HGVS-recommended cDNA numbering starts from the first ATG of the full coding sequence. Protein reference sequences should represent the ***primary translation product***, not a processed mature protein, and thus include any signal peptide sequences Currently many variants are present in the literature under several titles, and the correct identification of intron variants is especially problematic. No reliable overview of known *FH *variation can be achieved without a consistent nomenclature, which includes the appropriate numbering systems at both the DNA and protein level. We now recommend that all authors implement the recommendations of the HGVS when describing future *FH *variants.

The *FH *mutation database will accept all variants and if necessary assist in assigning the correct nomenclature. In light of the inconsistency of the recent *FH *mutation nomenclature, variants have been reassigned, where necessary, and an extra column has been generated clearly stating the originally reported description(s) of the mutation at the protein level, and where equivocal also at the cDNA level. Inclusion of sequence variants in the *FH *mutation database does not imply that there is convincing evidence for pathogenicity. Please refer to the disclaimer on the website.

Within the *FH *database, all variants that disrupt the reading frame, affect highly conserved residues (conserved by sequence alignment in at least *Saccharomyces cerevisiae*) or disrupt the consensus donor or acceptor splice sites (GT-AG), and are not found in healthy controls, should be suspected as potentially pathogenic. Non-conserved missense variants and potential splice site mutations that do not disrupt the consensus splice sites, are considered to be SNPs or rare variants of uncertain significance. Ideally, several lines of evidence should support publications which present mutations as "pathogenic", including screening DNA from a panel of 50–200 healthy individuals, describing the nature of the amino acid substitution (conservative/non-conservative) and the significance of the position in the protein (evolutionarily conservation or known functional domain). It is important to note whether the mutation has previously been reported, if it has been found in several families, and if it segregates with the disease within the family.

Many FH variants have been analysed for effects on enzymatic function, and this data has, in many cases, allowed the classification of missense variants as pathogenic. In addition a SIFT (Sorting Intolerant From Tolerant) analysis [34] of missense FH variants has been included. A high score (above 0.05) indicates that an amino acid is poorly conserved (alignment of 337 Uniprot proteins), indicating caution in assignment of function to the variant.

However none of these factors can be seen as definitive and each variant must be considered on its merits. Unfortunately, most mutations are currently reported without this accompanying analysis, and many have been identified in a single case or family. Thus caution should be exercised when attempting to derive clinically relevant information from the database, and users must carefully weigh all the evidence in the database and any additional data.

The *FH *gene is very highly conserved, showing 67% protein identity between humans and *S. cerevisiae *with the few coding SNPs described in dbSNP present in the HapMap population as very rare alleles (the minor allele being undetected). Thus, current knowledge suggests that most missense changes will be pathogenic. Polymorphisms, including intron variants, synonymous (silent) variants, nonsynonymous missense variants found in a healthy control panel and potential non-consensus splice site mutations but without evidence for transcript rearrangements are included in the database as such unless accompanied by clear evidence of pathogenic potential.

An abbreviated description of the FH related syndrome is given under "disease". Refer to the database notes for an explanation of these abbreviations. Further information can be found under "remarks", including the country of origin of the patient or study, together with other details such as the number of healthy controls tested for the variant, and any other supporting evidence.

## Utility

**Figure 1:**
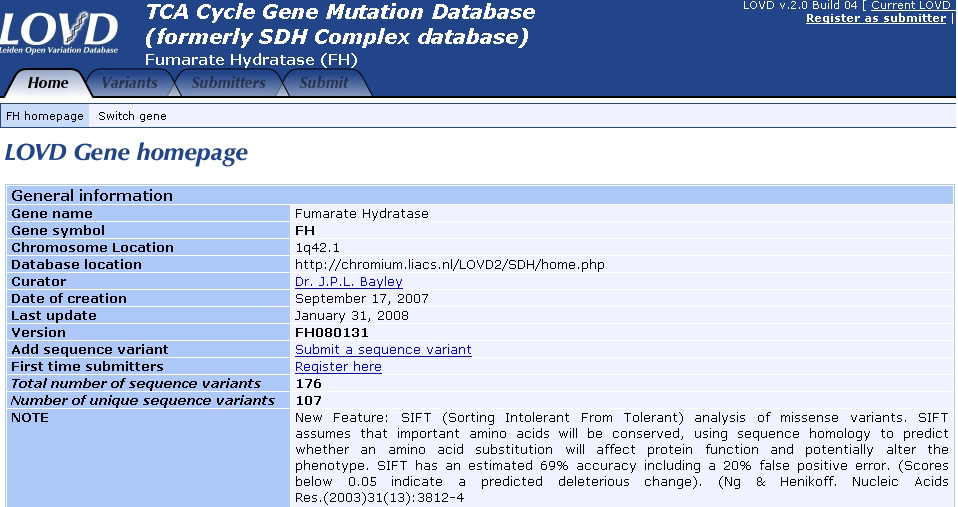
The *FH *mutation database introductory page. In addition to summary tables, various search options are also available.

The *FH *mutation database describes mutations exon by exon; giving a complete overview of all reported or directly submitted mutations in a single table. This overview permits the swift appraisal of the status of any new variant. The *FH *mutation database summary page lists general gene and database information and provides access to the tables containing the allelic variant information and several search options (Fig 1). In addition, the complete contents of the public database can be downloaded here and imported into a spreadsheet program for further analysis.

**Figure 2:**
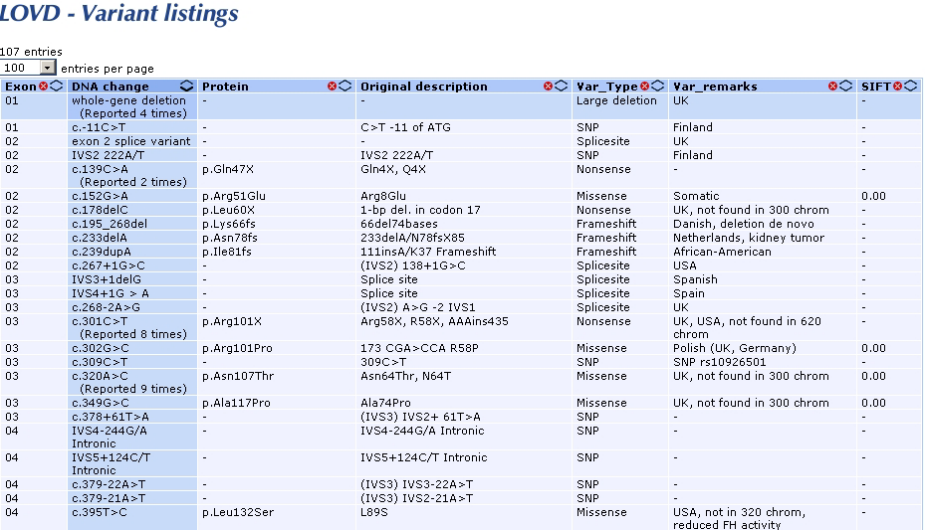
A partial overview of the *FH *unique allelic variants table.

The variant tables contain the sequence variants ordered by position, relative to the cDNA reference sequence (Fig 2). Tables describing both unique variants and all reported variants can be selected. Using the sort option in the column heading, variants can be re-ordered as required. An up-to-date and fully referenced overview of *FH *mutations will be especially useful to clinical geneticists, research scientists, and physicians involved in the care and treatment of patients with HLRCC or congenital fumarase deficiency.

## Discussion

**Figure 3:**
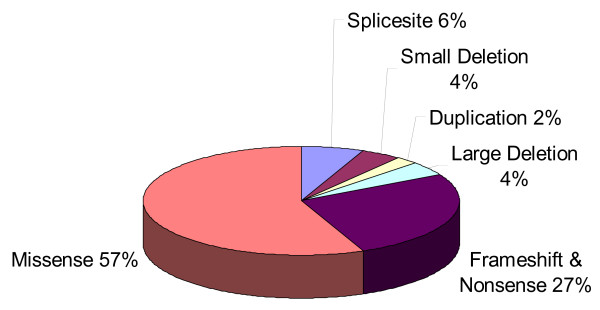
Summary of the relative frequency of *FH *variant types. Small deletions include all except whole exon deletions; large deletions include whole exon to whole gene deletions.

**Figure 4:**
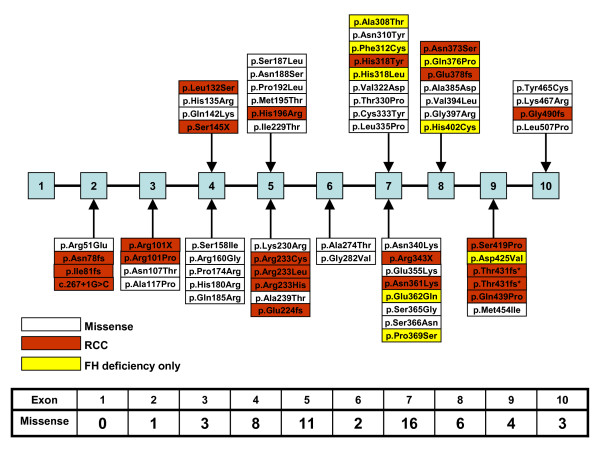
Overview of the exon distribution of *FH *missense, renal cell cancer associated and exclusively FH deficiency related mutations. Mutations in red have been identified in cases of renal cell cancer of either type II papillary or collecting duct morphology. Variants in yellow have (as yet) been found exclusively in cases of FH deficiency. The accompanying table lists the numbers of missense variants per exon. (*These mutations are distinct at the DNA level).

The *FH *database includes (as of Jan, 2008) 107 variants of which 93 are thought to be pathogenic and 14 may be non-functional intron variants. The most common types of mutations at the protein level are missense (57%), frameshifts and nonsense (27%), with the remainder composed of deletions, insertions and duplications (Fig 3). No mutation has yet been identified in exon 1 (often described as exon 0), and few large deletions have been reported but this probably reflects limited effort in this direction to date. The missense mutations are concentrated in several central exons of the gene, particularly 4, 5, 7 and 8. Unsurprisingly the most conserved exon is 7, followed by exon 4. It can also be noticed that the missense mutations of exon 5 cluster in the proximal and distal regions of the exon, which are both highly conserved (Fig 4).

That conservation is not always a good predictor of function is seen with Lys467Arg (K424R) missense variant. This amino acid is relatively poorly conserved, present in *E. coli*, but variable in many other species. The SIFT score is 0.17, indicating that this is a tolerated variant, and should not affect protein function. Other species variants include Asn, Ala, Met, and Glu, all of which are also predicted by SIFT analysis to be tolerated changes. However, Ylisaukko-oja *et al *recently demonstrated that this variant has less than half the activity of the wt variant [13]. Context may also be important for the function of a variant, a case in point being Glu362Gln (Glu319Gln), the very first FH mutation, described by Bourgeron in 1994 [2]. Two siblings with profound fumarase deficiency were found to be homozygous for this mutation, showing only residual fumarase activity of less than 0.5% of controls. The glutamine (Gln) variant residue is the only change predicted by SIFT analysis to be tolerated. It can be speculated that any other change in this highly conserved distal region of exon 7 would have resulted in such a complete loss of fumarase activity as to be incompatible with life.

Renal cell cancer (RCC) associated mutations occur throughout the gene with the exception of exons 1 and 6, though there are perhaps fewer RCC-associated mutations in exon 7 than one might expect (Fig 4). The Arg233His (R190H) mutation is the most commonly described *FH *variant, so it is unsurprising this residue should be mutated in a few cases of RCC. However, arginine 233 is also independently affected, with less common variants (Arg233Cys, Arg233Leu) and all changes have been associated with RCC. This residue resides in the A-site which may be the main catalytically active site [35]. Previously Alam *et al *[36] speculated that truncating mutations may be over-represented in renal cancer families. Within the current database, truncating and missense mutations are equally represented (11 vs. 11) in renal cell cancer patients (Fig 4). This still represents relatively more truncating mutations associated with RCC than in the overall database, but the current trend seems to indicate that the initial imbalance was an artefact of the limited numbers of variants then known. Whether truncating mutations are more penetrant, leading to more cases of RCC than the missense variants, is currently unclear from the published clinical data.

A number of *FH *missense mutations have been reported to reduce FH activity below that seen for truncating mutations, indicating a dominant negative action [37]. A second study failed to confirm differential activities of truncating and missense mutations on FH enzyme activity in lymphoblastoid cell lines from HLRCC patients [38]. However, Lorenzato *et al *recently formally demonstrated the dominant negative action of the Arg233His (R190H) mutation [39]. For a more complete functional analysis of *FH *missense mutations see Alam *et al *[36,37].

As described above, patients with mutations of *FH *display a range of pathologies, the most profound, FH deficiency, related to mutation of both *FH *alleles. We have previously noted [3,37] the tendency of FH deficiency-associated mutations to occur later in the gene (Fig 4) and to be less likely to include mutations leading to complete loss of protein function. While several cases of FH deficiency have been reported together with truncating mutations, the other affected allele tended to carry a missense mutation. The only case carrying two profound mutations was reported by Coughlin *et al *[40]. This patient (F1430) had only 2% residual FH enzyme activity, but did not show the most serious of the known clinical manifestations. Even the mutations in this patient may have been attenuated by the fact that while one allele was affected by a 74 bp deletion in exon 2, the other truncating mutation occurred very late in the protein at the end of exon 10, leading to the loss of the last 10 amino acid residues. These data suggest that some residual enzyme activity is required if an embryo is to develop to term.

## Conclusion

Here we present an online database of *FH *gene variants that provides the only complete and up-to-date overview of all reported disease-related mutations.

Clearly, considering the geographical distribution of the studies to date, only a fraction of all *FH *mutations associated with MCUL/HLRCC have yet been identified. While no unequivocal genotype-phenotype correlations associated with certain types of mutations or location in the gene have emerged so far [37], increasing knowledge of *FH *mutations may yet provide insight into sub-phenotypes or FH protein function. Importantly, every *FH *mutation found in an MCUL/HLRCC patient increases confidence in the pathogenic role of that variant, improving the accuracy of clinical genetic counselling. Equally it remains extremely important to test missense variants in sufficient, ethnically matched, healthy controls to aid in identifying SNPs in the coding sequence, which may currently be incorrectly classified.

We hope that the *FH *mutation database, which strives to systematically unify all current genetic knowledge of *FH *variants, will increase the confidence of clinical geneticists and treating physicians when advising patients and their families, will provide a convenient resource for research scientists, and may eventually assist in gaining novel insights into FH and its related clinical syndromes.

## Availability and requirements

The *FH *mutation database;  is freely accessible and all researchers may submit new sequence variants online (after registration – to collect contact information for reference purposes and clarification of submitted details, as well as to assign a login name and password).

## Competing interests

The author(s) declare that they have no competing interests.

## Authors' contributions

JPB carried out the analysis and editing of data, wrote the manuscript, and is the principal database curator. IPMT collected, edited and analysed these data, and co-wrote the manuscript. VL collected, edited and analysed these data and co-wrote the manuscript. All authors read and approved the final manuscript.
